# Braf-Mutant Melanomas: Biology and Therapy

**DOI:** 10.3390/curroncol31120568

**Published:** 2024-12-03

**Authors:** Elvira Pelosi, Germana Castelli, Ugo Testa

**Affiliations:** Department of Oncology, Istituto Superiore di Sanità, Viale Regina Elena 299, 00161 Rome, Italy; elvira.pelosi@iss.it (E.P.); germana.castelli@iss.it (G.C.)

**Keywords:** skin tumors, melanoma, BRAF mutations, MAPK, immunotherapy, targeted therapy, genomic profiling, tumor evolution

## Abstract

The incidence of melanoma, the most lethal form of skin cancer, has increased mainly due to ultraviolet exposure. The molecular characterization of melanomas has shown a high mutational burden led to the identification of some recurrent genetic alterations. *BRAF* gene is mutated in 40–50% of melanomas and its role in melanoma development is paramount. *BRAF* mutations confer constitutive activation of MAPK signalling. The large majority (about 90%) of *BRAF* mutations occur at amino acid 600; the majority are *BRAF^V600E^* mutations and less frequently *BRAF^v600K, V600D^* and *^V600M^*. The introduction of drugs that directly target *BRAF*-mutant *protein (BRAF inhibitors)* and of agents that stimulate immune response through targeting of immune check inhibitor consistently improved the survival of melanoma *BRAF^V600^*-mutant patients with unresectable/metastatic disease. In parallel, studies in melanoma stage II-III patients with resectable disease have shown that adjuvant therapy with ICIs and/or targeted therapy improves PFS and RFS, but not OS compared to placebo; however, neoadjuvant therapy plus adjuvant therapy improved therapeutic response compared to adjuvant therapy alone.

## 1. Introduction

Melanoma is an aggressive neoplasia that originates from the malignant transformation of melanocytes. It is responsible for most deaths related to skin tumors. Melanoma is a heterogeneous disease at both the phenotypical and molecular levels. The 2018 World Health Organization (WHO) classification of skin tumors identified nine different types of melanomas, differentiated for their epidemiology, clinical features and genomic alterations [1]. At the etiological level, melanomas can be distinguished into two large groups: one related to sun exposure and the other not related to sun exposure. This distinction is related to the presence or not of a UV-related mutational signature and to anatomical location. Sun-exposed melanomas are further subdivided into two subgroups according to the degree of cumulative solar damage (CSD): low-CDS melanomas, including superficial spreading melanomas (SSM), and high-CDS melanomas, including lentigo maligna and desmoplastic melanomas. The melanomas not related to sun exposure include acral melanomas, Spitz melanomas, mucosal melanomas and uveal melanomas [1].

Numerous studies have characterized the molecular abnormalities observed in cutaneous melanomas. These studies have shown that cutaneous melanomas are characterized by recurrent genetic alterations occurring at the level of genes involved in the RAS/MAPK/pathways (*BRAF*, *RAS*, *NF1*), telomerase (*Telomerase Promoter*), the cell cycle (*RB1*, *CDKN2A*), apoptosis (*TP53*, *MDM2*), the PTEN/PI3K/AKT pathway (*PTEN*, *PI3K)* and MITF (*MITF*) [2,3].

In 2015, a multiplatform analysis of a large cohort of cutaneous melanomas (mostly metastatic tumors) allowed the definition of four molecular subtypes, characterized by a distinct profile of genetic alterations: (a) the BRAF-mutated subtype (52% of total), with frequent hot-spot mutations mostly occurring at the level of V600, and more rarely of V601, and rare non-spot mutations at the level of exon 11; hot-spot *BRAF* mutations are usually mutually exclusive with *RAS* mutations, while exon 11 *BRAF* mutations co-occur with *RAS* hot-spot and *NF1* mutations; (b) the RAS-mutated subtype (about 30% of total), characterized by RAS genes mutating much more frequently than *HRAS* or KRAS; (c) the NF1-mutated subtype (14% of total), characterized by loss-of-function *NF1* mutations leading to MAPK activation; and (d) the triple-wild-type subtype (15% of total), characterized by an absence of hot-spot *BRAF*, *RAS* and *NF1* mutations and by the presence of *KIT*, *GNAQ* and *TYRP1* mutations in 10–20% of cases [4].

Superficial spreading melanoma (SSM) and nodular melanoma (NM) represent the two most common primary melanoma histologic subtypes, accounting for about 70% and 15% of cases. While SSM is characterized by a radial phase of tumor growth followed by a vertical phase of growth with cutaneous invasion and then metastases, NM rapidly moves to the vertical growth phase, with no horizontal phase of growth. The peculiarities of NMs are represented by their thickness, their development in body areas exposed to sun and their rapid time of development (within weeks or a few months) [5]. NM is the deadliest melanoma subtype and is responsible for about 40% of melanoma deaths [5]. *BRAF* is the most common gene mutation observed in NM; *NRAS* mutations, upregulated PD-L1 expression and low lymphocyte infiltration are typical features of NM [5]. Ribosomal protein S6 kinase polypeptide 1 (RSK1) seems to be specifically activated in NM and promotes tumor invasion [6].

Non-cutaneous melanomas display some remarkable differences in their genetic abnormalities compared to cutaneous melanomas. Acral melanomas display a profile of driver mutations similar to that observed in cutaneous melanomas, with *BRAF* being the most frequently mutated, followed by *NRAS* and *NF1* mutations. *TERT* promoter mutations are less frequent than in cutaneous melanomas. Acral melanomas have a higher frequency of triple-negative melanomas (45–58%) and consequently exhibit a higher frequency of *KIT*, *GNAQ* and *TYRP1* mutations compared with cutaneous melanomas [2,3].

Mucosal melanomas have a mutational profile characterized by *KIT* as the most frequent mutations (20–25% of cases), followed by *BRAF* and *NRAS* mutations (whose frequency is lower than that observed in cutaneous melanomas) and *NF1* mutations. *TERT* promoter mutations are observed at lower frequency than in cutaneous melanomas. The mutational burden is lower in mucosal melanomas than in cutaneous melanomas [2,3].

Uveal melanomas are the most frequent tumors of the eye and display a genomic mutational profile different from cutaneous melanomas, with *GNAQ/GNA11*, *BAP1* and *SF3B1* being the genes most frequently mutated [2,3].

## 2. BRAF-Mutated Melanomas

The mitogen-activated protein (MAP) signaling pathway, also known as RAS/RAF/MEK/ERK, regulates cell proliferation through a cascade of kinase phosphorylation. The process is initiated by the binding of an extracellular ligand to a mitogenic transmembrane receptor, with consequent receptor dimerization and autophosphorylation and GDP conversion to GTP; then, the GTP-RAS complex activates BRAF, which forms dimers and activates the cascade involving MEK 1-2 and ERK 1-2, leading finally to the activation of cell proliferation. BRAF is therefore a key serine/threonine protein kinase of the MAPK pathway. The BRAF protein is encoded by the *BRAF* gene, also known as proto-oncogene B-Raf and v-Raf murine sarcoma viral oncogene homology B.

**Figure 1:**
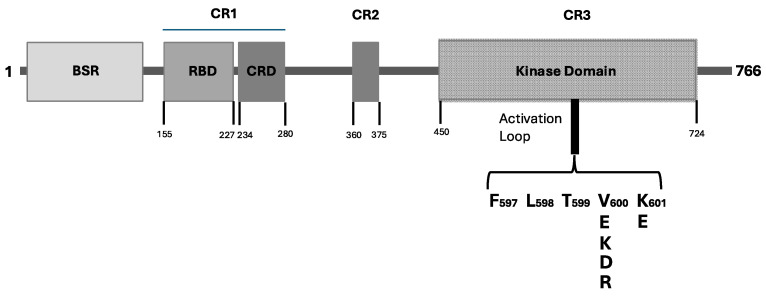
Schematic representation of the structure of BRAF protein. BRAF protein is composed of 766 amino acids. Several functionally relevant structural regions are identified: BSR (BRAF-specific region); CR1 (Constant Region 1) containing two domains, RBD (Ras-binding domain) and CRD (Cystein-Rich Domain); CR2 (Constant Region 2); CR3 containing the kinase domain. Within the kinase domain, the activation loop is outlined with the most frequent missense mutations observed in melanomas.

The BRAF protein belongs to the Raf kinase family of growth signal transduction kinases and is composed of 766 amino acids. This protein is composed of three conserved domains (CR) typical of the Raf kinase family members: conserved region 1 (CR1) contains a GTP-Ras, an autoregulatory binding domain; conserved region 2 (CR2) is a serine-rich hinge region; and conserved region 3 (CR3) contains a catalytic protein kinase domain involved in the phosphorylation of substrates at the level of specific consensus sequences. Particularly, the CR1 region exerts an auto-inhibitory activity on BRAF activation through the inhibition of the CR3 kinase domain; this region contains two subdomains: a Ras-binding domain (RBD), located at 155–227 and involved in GTP-Ras binding, and a Cystein-rich domain (CRD), a phorbol ester/DAG-binding zinc finger motif located at 234–280 and involved in B-Raf membrane docking after Ras binding. An initial N-terminal domain, called BRAF-specific region (BSR) and located at 10–145, together with CRD exerts an inhibitory regulation of BRAF activation [7]. CR2 is a flexible linker region that contains a Raf phosphorylation site and a binding site for the 14-3-3 protein, which contributes to maintaining Raf in its autoinhibited state [8]. CR3 contains the BRAF enzymatic domain located at 457–717 and is subdivided into two lobes connected by a short hinge region: the N-lobe located at at 457–530 is responsible for ATP binding; the C-lobe located at the active site is represented by a cleft located between the two lobes, and the catalytic site involves the Asp residues at 576 in the C-lobe, located facing the cleft [9]. CR3 contains some subregions playing an important role in enzyme physiology; furthermore, some of these subregions are altered in their function as result of cancer-related genetic alterations. The P-loop is located at residues 467–471 and is involved in the stabilization of the non-transferable phosphate groups of ATP during enzymatic ATP binding. The catalytic loop at residues 574–581 contributes to the enzymatic activity of the kinase domain, assisting in the transfer of the γ-phosphate of ATP to the BRAF protein substrate. The activation loop is located at the level of residues 596–600 and is strongly bound to the P-loop through hydrophobic interactions, thus triggering the shift in the enzyme to its active state. (Figure 1).

### 2.1. BRAF Mutations

*BRAF* is frequently mutated in several human cancers. Mutations in melanoma and thyroid cancer are particularly frequent, while mutations in colorectal cancer, non-small cell lung cancer, glioma and bladder cancer are less frequent [10]. *BRAF* mutations occurring in cancer are classified into four distinct molecular groups: class I mutations determine a high activation of Ras-independent monomeric BRAF (all these variants are missense mutations of Val600, an amino acid residue located in the activation loop); class II *BRAF* mutants signal constitutively active RAF dimers and determine a moderate or high Ras-independent activation of BRAF; class III *BRAF* mutations determine the RAS-dependent activation of home and heterodimers and generate a loss-of-function BRAF variant; class IV mutations are related to fusion events involving the *BRAF* gene [10].

In melanomas, the most frequent mutations are represented by class I mutations (about 75%), followed by class I and class III mutations (about 12% and 11%, respectively). Among class I mutations, the most frequent mutations are represented by V600E mutations, caused by a missense mutation determining the substitution of a Valine residue with a Glutamine residue. V600E mutants exhibit strong BRAF activity, estimated to be about 500 times higher than WT-BRAF; this mutant disrupts the interaction between the activation loop and the P-loop and thus determines the constitutive BRAF activation. The high BRAF activation directly signals to ERK through the phosphorylation of MEK [11]. The second most common V600 mutant is V600K, generated by a missense mutation leading to the substitution of Valine 600 with Arginine; V600K melanomas are usually observed in patients with a history of chronic sun damage and exposure and are associated with a poor prognosis. V600K melanomas are less dependent on the MAPK/ERK pathway, with a higher expression of PI3KB [12,13]. *BRAF V600K*-mutated melanomas have dermoscopic features identical to those observed in *BRAF V600E*-mutated melanomas; furthermore, both groups have a nodular appearance [13]. Other studies have shown that *BRAF* mutational status cannot be effectively predicted based on early dermoscopic features [14].

The V600R variant, involving the substitution of Valine with Arginine, and V600D, involving the substitution of Valine with Aspartic acid, are more rarely observed in melanoma patients and display biological characteristics like those reported for the V600K variant.

The second generation of ATP-competitive RAF inhibitors called class I inhibitors, including vemurafenib, dabrafenib and encorafenib, are potent and selective inhibitors of BRAF^V600^ mutants [15]. These inhibitors, in addition to exerting a potent inhibition of BRAF^V600^ mutants, also induce a paradoxical activation of RAF signaling in BRAF-WT cells and tumors, including those with *RAS* mutations; this phenomenon is related to the differential effects of class I inhibitors on BRAF monomers (inhibitory) versus BRAF dimers (stimulatory) [16]. It is important to underline that the RAF inhibitor paradox also represents the molecular basis for the clinical success of regimens based on the association of a BRAF inhibitor with an MEK inhibitor; in fact, the early ERK activation induced by class I BRAF inhibitors through the RAF inhibitor paradox is inhibited by MEK inhibitors.

As above discussed, class II mutations are less activating than class I mutations and can be subdivided into class IIa and IIb, according to their mutational profile, with L597 and K601 mutations of the activation loop observed in class IIa and G476 and G469 of the glycine-rich region of the kinase domain observed in class IIb mutations [17]. Melanomas bearing class II mutations are less sensitive to BRAF inhibitors than those with class I mutations; however, class II-mutant melanomas are sensitive to double inhibition with BRAF and MEK inhibitors [18]. Analyses of the literature data have further supported the conclusion that melanomas bearing class II mutations are more sensitive to the combination of BRAF plus MEK inhibitors than to BRAF or MEK inhibitors alone [19].

Class III *BRAF* mutations have a different mutational profile involving N581 or D594 residues and are RAS-dependent, have low or absent BRAF kinase activity and cooperate with concurrent *RAS* or *NF1* mutations; melanomas bearing these mutations are less sensitive to BRAF+MEK inhibitors than melanomas with class II mutations [17].

An additional event that can induce BRAF activation is represented by *BRAF* fusion events, observed in a minority of melanoma patients; these fusion events are usually not associated with *BRAF* mutations and are highly heterogeneous at the molecular level due to the variability of *BRAF* fusion partners [20,21]. This molecular heterogeneity is also reflected by a concomitant phenotypic and clinical heterogeneity, with variable responses to BRAF/MEK inhibitors [22].

Co-mutational analysis showed that six different genes have mutual exclusivity with *BRAF*, including the driver genes *NRAS* and *NF1*, in line with their complementary roles in the activation of the MAPK pathway; similarly, *TLR4* and *EGFR* are both able to regulate the MAPK pathway and are also mutually exclusive with the *BRAF* mutation. In addition, the *ARH*, *GAP21* and *GABRA 6* genes are also mutually exclusive with *BRAF* mutations [23].

The co-mutation profile of class III *BRAF*-mutant melanomas differs substantially from that observed in class I *BRAF*-mutant melanomas. In fact, *NF1* loss-of-function mutations frequently co-occur with *BRAF* non-V600 class II mutations; in fact, melanomas harboring *BRAF* III non-V600 mutations have frequently co-occurring *NF1* loss-of-function mutations (67%) and *RAS* mutations (22%). Furthermore, 15% of *NF1* loss-of-function mutant melanomas harbor concomitant class III C600 *BRAF* mutations [24].

In some melanoma patients, *BRAF* copy number gains may be observed in addition to *BRAF* mutations. The *BRAF* gene is located on chromosome 7q and the gains of chromosome 7 are observed in about 50% of patients with primary cutaneous melanomas [25]. Maldonado and coworkers reported that 9 patients out of 19 with *BRAF* mutations had an increased 7q number; on the other hand, 15 patients out of 49 with WT-*BRAF* melanomas exhibited an increased 7q number [26]. In seven of the nine *BRAF*-mutant patients with 7q gain, *BRAF* allelic ratio evaluation suggested a gain of the mutant *BRAF* allele [26]. Helias-Rodzewicz et al. evaluated variations in *BRAF*-mutant allele percentage and 7q copy number in 368 melanoma patients [27]. Of these patients, 38% displayed *BRAF* mutations, 66% of which showed heterozygous allele frequency and 19% *BRAF*-mutant allele frequency >60%; chromosome 7 polysomy was observed in 16% of *BRAF*-WT, 35% of *BRAF*-heterozygous and 54% of *BRAF*-mutant cases with allelic frequency >60% [27]. In parallel, 33 melanocytic nevi were explored, with 27 displaying V600E *BRAF* mutations, all without chromosome 7 gain [27]. Comparable observations were made by Stagni et al., who investigated 46 metastatic *BRAF*-mutant melanoma patients before treatment with *BRAF* inhibitors; 50% displayed *BRAF* gains, mostly related to chromosome 7 gain. The analysis of *BRAF*-mutant allele frequency showed that 64% of cases were heterozygous, while 12.5% and 23% of them showed low and high *BRAF*-mutant allele frequency; patients with high *BRAF*-mutant allele frequency have concomitant *BRAF* copy number gain [28]. Importantly, patients with heterozygous or high *BRAF*-mutant allele frequency responded to treatment with BRAF inhibitors better than those with low *BRAF*-mutant allele frequency [28].

Birkeland and coworkers have explored the patterns of genomic evolution occurring in advanced melanoma at the level of metastatic progression. An event observed in many melanoma patients at the advanced stage consisted of a low level of copy number gains of at least one *BRAF*-containing allele, occurring in 78% of patients with *BRAF*-mutant melanomas and in 15% of *BRAF*-WT melanomas; in all patients with *BRAF*-mutant melanomas, with only one exception, the gained *BRAF* allele was the mutated allele [29]. Whole-genome duplication was an event observed in about 40% of patients and occurred later than *BRAF* copy number gain [29].

### 2.2. Role of BRAF Mutations in Melanomagenesis

Many studies have supported a key role of *BRAF* mutations in the early stages of melanomagenesis. First, *BRAF* mutations, such as *BRAF*^V600E^*,* were initially reported in 82% of melanocytic nevi cases, with *N-RAS* mutations present in a minority of cases [30]. This finding was confirmed in subsequent studies [31]. The *BRAF* mutation involves one of the two alleles in every melanocytic nevus, thus suggesting that a nevus derives from the outgrowth of a single melanocyte that acquires *BRAF* mutations [32]. This interpretation is directly supported by the immunostaining of melanocytic nevi with an antibody (VE1) specific for *BRAF*^V600E^ mutation, resulting in the detection of this genetic abnormality in all melanocytes comprising a *BRAF*-mutated nevus [32]. The key role of *BRAF* mutations in nevi formation is directly supported by studies on animal models engineered to express *BRAF*^V600E^ in the melanocyte lineage (transgenic mice expressing *BRAF*^V600E^ under the control of the melanocyte-specific *mtfa* promoter). These animals developed patches of ectopic melanocytes (“fish nevi”) [33]. In *TP53*-deficient fish, *BRAF*-mutant-generated nevi progressed to melanomas [33].

The sequencing of melanoma-relevant genes in primary melanomas and their adjacent precursor lesions, including benign nevi areas and intermediate lesions to the melanoma, allowed researchers to define the genetic evolution from nevi to melanomas [34]. Benign nevi lesions harbored *BRAF*^V600E^ mutations, while intermediate lesions were enriched in *NRAS* and *BRAF*^V600E^ mutations and additional driver mutations; intermediate lesions and melanomas in situ harbored *TERT* promoter mutations, while biallelic *CDKN2A* inactivation, as well as *PTEN* and *TP53* mutations, emerged only in advanced melanomas [34]. A sequential genomic and transcriptomic analysis extending from nevi up to regional metastases allowed the definition of pathways activated or disrupted during melanoma evolution; somatic alterations sequentially induced NAPK pathway activation, the upregulation of telomerase, the modulation of the chromatin landscape, G1/S checkpoint override, the ramp-up of MAPK signaling, the disruption of the *p53* pathway and the activation of the PI3K pathway [35].

The study by Shain and coworkers provided evidence that copy number alterations represent a genetic abnormality mainly observed at late stages of melanoma evolution. Matsuta and coworkers observed chromosome 7 gains in 41% of a melanoma population mainly composed by primary melanomas [36]. Casorzo et al. observed the absence of chromosome 7 polysomy in melanocytic nevi; in nevus-associated melanomas, chromosome 7 gains were observed only in melanoma sectors of these lesions [37]. Udart et al. observed a higher frequency of chromosome 7 polysomy in metastatic melanomas compared to primary melanomas (41% vs. 15%, respectively) [38].

## 3. Therapy of BRAF-Mutant Melanomas

The progress made in understanding the genetic alterations of melanomas has led to the development of numerous new drugs that have been introduced and evaluated in clinical trials.

Concerning *BRAF*-mutant melanomas, two types of new drugs were introduced in therapy in recent years: one represented by drugs directly and specifically targeting either *BRAF*-mutant molecules or MEK, and the other one represented by immune check inhibitors—drugs targeting molecules that negatively regulate the immune response. The introduction of these drugs has completely revolutionized the medical therapy of *BRAF*-mutant melanomas.

### 3.1. Adjuvant Therapy in Stage II Melanoma

It is estimated that about 15% of newly diagnosed melanomas are stage II tumors; about 7% of all melanomas are stage IIB and IIC melanomas, with an infiltration of the dermis of >2 mm or >4 mm, respectively, and with a consistent risk of recurrence. Outcomes for patients with stage II melanomas are highly heterogeneous, with a very low risk of death for patients at stage IIA (94% survival at 10 years) to a moderate risk of death for patients at stage IIB (85% survival at 10 years) and at stage IIC (75% survival at 10 years). Thus, there is a clear rationale for an adjuvant therapy for resected stage IIB/C melanoma patients. Obviously, this therapeutic choice has to take into account a risk/benefit analysis.

Two pivotal clinical trials have evaluated the safety and effectiveness of adjuvant pembrolizumab and adjuvant nivolumab in resected stage IIB/C melanoma patients (Table 1). The KEYNOTE-716 phase III randomized, double-blind trial explored the safety and effectiveness of pembrolizumab in 976 melanoma patients, resected, with stage IIB/C cutaneous melanoma without lymph node regional involvement, randomly assigned to treatment with pembrolizumab (487 patients) or to placebo (489 patients) [39]. The main endpoint of the study consisted of evaluation of the effect of treatment on RFS and DMFS. Median RFS was not reached in either group; the estimated 36-month RFS rate was 76.2% for pembrolizumab and 63.4% for the placebo. In both stage IIB (79.7% vs. 66.5%, respectively) and IIC (71.4% vs. 58%, respectively), an improvement in RFS in the pembrolizumab group compared to the placebo group was observed [39]. Median DMFS was not reached in either group; the estimated 36-month DMFS was 84.4% for pembrolizumab and 74.7% for the placebo [36]. The improvement of DMFS was observed in both stage IIB (86.7% vs. 78.9%) and stage IIC patients (80.9% vs. 68.1%, respectively) [39]. The CHECK Mate 76K phase III, double-blind trial involved the enrollment of 790 melanoma patients with stage IIB/C disease, randomized 2:1 to treatment with nivolumab or with a placebo. At 7.8 months of follow-up, nivolumab improved both RFS and DMFS over the placebo [40]. At 12 months, RFS was 89% among patients treated with nivolumab compared to 79% in the placebo group; this improvement was related to a reduction in distant (4.9% vs. 11.7%) and locoregional (2.1% vs. 7.6%) recurrences compared to the placebo. The RFS benefit due to nivolumab was observed in both stage IIA and IIB patients [40]. Importantly, nivolumab improved RFS both in *BRAF*-WT (91.2% vs. 77.1%) and *BRAF^V600^*-mutant melanomas (87.3% vs. 81.7%) [40]. Furthermore, nivolumab improved DMFS rate compared to the placebo: at 12 months, DMFS was 92.3% in the nivolumab group compared to 86.7% in the placebo group [40]. This improvement in DMFS was particularly evident among stage IIC patients (87.9% vs. 78.7%) [40]. Treatment-related grade 3-4 adverse events were observed in 10.3% (nivolumab) and 2.3% (placebo) of patients [40]. One treatment-related death (0.2%) occurred in patients treated with nivolumab.

Recently, a phase III clinical trial was proposed aiming to evaluate the safety and efficacy of a combined BRAF+MEK inhibitor (encorafenib+binimetinib) in resected stage II *BRAF^V600^*-mutant melanoma patients [41].

Interestingly, the phase II/III randomized trial DETECTION will explore circulating tumor DNA-guided therapy for stage IIB/IIC melanoma patients after surgical resection [42]. This clinical trial is based on several recent studies showing that circulating tumor DNA (ctDNA, the tumor-derived fraction of circulating free DNA in the blood) represents a suitable biomarker of active tumor disease in stage II/III melanoma patients [43,44]. A recent study based on the analysis of 90 stage IIA-IIID cutaneous melanoma patients showed that personalized, tumor-informed ctDNA analysis offers an additional tool to conventional monitoring for melanoma patients to detect molecular residual disease and was found to be prognostic [45]. Furthermore, the longitudinal monitoring of ctDNA using a personalized assay is highly prognostic in stage II/III melanoma patients undergoing curative resection [46]. Thus, the DETECTION trial will involve melanoma patients with stage IIB/IIC disease with *BRAF/NRAS/TERT promoter* mutations, surgically resected; the patients will be analyzed for the presence of ctDNA, and those positive will be randomized 1:1 to continue routine follow-up and therapy with either the clinical option under investigation or treatment with nivolumab [47].

### 3.2. Adjuvant Therapy in Stage III Melanoma

The immunotherapy randomized phase III trial EORTC 18071 showed a better RFS and OS of stage III melanoma patients treated with ipilimumab compared with the placebo [44]. In the evaluation, with 7 years of follow-up, RFS (HR 0.75, *p* < 0.001), DMFS (HR 0.76, *p* < 0.002) and OS (HR 0.73, *p* < 0.002) showed a significant benefit in the ipilimumab group compared with the placebo group [48].

These results have supported the approval of ipilimumab as an adjuvant therapy for stage III melanoma by the US FDA. However, the high toxicity of ipilimumab monotherapy greatly limits its current adoption in adjuvant settings. In the EORTC 1325/KN-054 trial (phase III, double-blind), high-risk stage IIIA/B surgically resected melanoma patients were randomly assigned to receive 200 mg of pembrolizumab or placebo intravenously every 3 weeks for a total of 18 doses (about 1 year). A five-year analysis of this study was reported in 2022 [46]. In the overall treated population, pembrolizumab administration was associated with longer recurrence-free survival (RFS) than the placebo (55% vs. 39%, respectively) and longer metastasis-free survival (MFS) than the placebo (60% vs. 44%, respectively) [49]. In patients with *BRAF-V600* mutations, the RFS at 5 years was 54% in the pembrolizumab group and 35% in the placebo group [49]. Adverse events were rarely observed and limited to nine patients among those treated with pembrolizumab and one patient treated with the placebo [49]. An analysis extended to seven-year follow-up confirmed the results observed in the previous analysis. Particularly, at 7 years, DMFS was 54% in the pembrolizumab group and 42% in the placebo group; progression/recurrence-free survival 2 (PRSF2) was 61% in the pembrolizumab group and 53% in the placebo group [50].

The randomized phase III Check Mate 238 study compared the safety and the efficacy of nivolumab and ipilimumab in 906 melanoma patients with stage IIIB, IIIC, or IV tumors surgically resected [51]. The initial results observed in this study showed a 12-month RFS of 70.5% in the nivolumab group and 60.8% in the ipilimumab group [51]. Furthermore, a lower rate of grade 3–4 adverse events was observed in the nivolumab group compared to the ipilimumab group [51]. Both patients with *BRAF*-WT and *BRAF*-mutant melanomas displayed benefit from nivolumab therapy [51]. According to these results, nivolumab was approved by the US FDA in 2017. The analysis of cancer recurrence in patients involved in this trial showed a rate of recurrence of 44% in the nivolumab group and 51% in the ipilimumab group [52]. Nivolumab-treated patients with early or late recurrence and *BRAF*-mutant tumors benefitted from an ipilimumab-based therapy or targeted therapy (BRAF and MEK inhibitors) [52]. In both groups of patients, biomarkers associated with improved RFS and OS were represented by higher levels of tumor mutational burden, tumor PD-L1, intratumoral CD8^+^ T cells and IFN-γ-associated gene signatures, and lower levels of serum C-reactive protein [53].

The randomized phase III trial SWOG 1404 evaluated whether adjuvant pembrolizumab (647 patients) improved RFS or OS in comparison with high-dose IFNα-2b for one year or ipilimumab for up to three years (654 patients) in resected high-risk stage III melanoma patients [54]. At a median follow-up of 47.5 months, pembrolizumab was associated with significantly longer RFS compared to the IFNα-2b and ipilimumab treatments (HR 0.77, *p* < 0.002); however, pembrolizumab did not improve OS compared to the other two therapies [54]. Treatment-related adverse events (grade 3–5) were lower in patients treated with pembrolizumab (19.5%) than in those treated with IFNα-2b (71.2%) or ipilimumab (49.2%) [54]. A secondary analysis showed that quality of life was significantly better among patients treated with pembrolizumab compared to the two immunotherapy groups [55].

The IMMUNED phase II randomized clinical trial compared adjuvant nivolumab (1 mg/kg) plus ipilimumab (3 mg/kg) versus nivolumab versus placebo in 167 patients with resected stage IV melanoma [56]. Four-year RFS was 64.2% in the nivolumab plus ipilimumab group, 31.4% in the nivolumab group and 15% in the placebo group; 4-year OS was 83.8% in the nivolumab plus ipilimumab group, 72.6% in the nivolumab group and 63.1% in the placebo group [56]. Interestingly, patients with *BRAF^V600^* mutations benefitted from nivolumab plus ipilimumab more than *BRAF*-WT patients (HR 0.11 vs. 0.44, *p* < 0.019). Grade 3–4 adverse events were more frequent in the nivolumab plus ipilimumab group (71% vs. 29%, respectively) [56].

However, the CheckMate 915 phase III double-blinded trial failed to show a benefit in RFS in patients receiving ipilimumab (1 mg/kg for 6 weeks) plus nivolumab (240 ng every two weeks) or nivolumab alone [57]. Probably, the lower dose of ipilimumab adopted in this study may explain the different results observed in this study compared to the IMMUNED trial [57].

In *BRAF*-mutant surgically resected melanoma stage III patients, the safety and efficacy of BRAF inhibitors associated with an MEK inhibitor were explored. Thus, the COMBI-AD trial (NCT 01682083) evaluated 12 months of treatment based on adjuvant therapy with dabrafenib plus trametinib or with placebo in patients with resected stage III melanoma with *BRAF* V600 mutations [55]. Particularly, in this double-blind, placebo-controlled, phase III trial, 870 patients with completely resected stage III melanoma with *BRAF*^V600E^ or *BRAF^V600K^* mutations received dabrafenib at a dose of 150 mg twice daily plus trametinib at a dose of 2mg once daily (combination therapy, 438 patients) or placebo (432 patients) for 12 months [58]. Based on the results observed for RFS at the prespecified date, the use of dabrafenib plus trametinib for stage III melanoma patients with *BRAF^V600^* mutations was approved in many countries. Recently, the final results of this trial were reported with a follow-up of more than 8 years [59]. Relapse-free survival and distant metastasis-free survival were significantly better for dabrafenib plus trametinib compared to the placebo; the analysis of overall survival showed that the risk of death was 20% lower in the dabrafenib plus trametinib group compared to the placebo, but this benefit was not significant. Among patients with *BRAF^V600E^* mutations, a 25% lower risk of death was observed in the dabrafenib plus trametinib group compared to the placebo, while in patients with *BRAF^V600K^* mutations, the overall survival was slightly better in the placebo group than in the dabrafenib plus trametinib group [59]. The interpretation of the results observed on overall survival is complex in that 42% of patients in the combination therapy group and 57% in the placebo group received additional anticancer treatments; among the patients who received systemic anticancer therapies after the discontinuation of dabrafenib and trametinib, BRAF-targeted therapy was used in 51% of the patients in the combination therapy group and 63% of those in the placebo group, as well as immunotherapy in 70% and 52%, respectively [59]. Varying responses to subsequent anticancer therapies were seen in the two trial groups. Particularly, patients in the placebo group responded better to immunotherapy than combination therapy (mOS not reached vs. 104 months, respectively), while the response to targeted therapy was similar in the two groups of patients. In the combination therapy group, median overall survival was longer among patients treated with immunotherapy (104.6 months) compared to those who received subsequent BRAF-targeted therapy (41.8 months) [59]. It is of interest to note that in the final results of the COMBI-AD trial, a subanalysis of OS and not RFS was reported according to the disease-stage subgroups; this subanalysis showed a trend of efficacy of dabrafenib plus trametinib for stage IIIB, IIIC and IIID, but not for IIIA [59]. An analysis of biomarkers correlated with response in these patients showed the following: MAPK pathway genomic alterations at baseline did not affect treatment benefit or clinical outcome; an IFN-γ gene expression signature higher than the median was prognostic for prolonged RFS in both treatment groups; and low tumor mutational burden was associated with longer RFS in the group of patients treated with dabrafenib and trametinib [60].

Patients with stage IIIA melanoma have been underrepresented in clinical trials of adjuvant therapy. Information deriving from the COMBI-AD and KEYNOTE-A54 trials failed to show any significant effect of dabrafenib plus trametinib or pembrolizumab, respectively, on RFS compared to the placebo in stage IIIA melanoma patients. A retrospective, multicentric trial involving adjuvant treatment showed that anti-PD1 or dabrafenib plus trametinib failed to improve RFS and DMFS compared to the placebo [61].

Adjuvant BRAF/MEK inhibitors and immunotherapy with anti-PD1 have become the standard of care for resected high-risk stage III melanoma patients. However, few studies have directly compared hand-to-hand BRAF/MEK inhibitors vs. anti-PD1 agents in resected *BRAF*-mutant melanoma patients (Table 2). In this context, a recent study based on a nation-wide cohort in the Netherlands allowed a comparison of the outcomes of all resected high-risk stage III melanoma patients treated with first-line BRAF/MEK inhibitors and anti-PD1, including 225 patients treated with BRAF/MEK inhibitors and 729 treated with anti-PD1 [62]. Through propensity score matching, two similar groups of 213 patients were defined; after matching, the 1- and 2-year RFS, DMFS and OS rates were not significantly different between the two groups of patients [58]. These observations suggest similar outcomes between adjuvant BRAF/MEK inhibitors and anti-PD1 treatment in stage III melanoma [62].

A multicenter, retrospective cohort study, performed in 15 melanoma centers located in various countries, involved the evaluation of outcomes of 598 melanoma patients with resected stage III *BRAF^V600^*-mutant melanoma undergoing treatment based on either anti-PD1 agents (205 patients) or dabrafenib plus trametinib (393 patients). At a median follow-up of 33 months, the median RFS was 51 months in the dabrafenib plus trametinib group and 44.8 months in the anti-PD1 group, with comparable OS and DMFS rates [63]. Among patients who experienced recurrence, the proportion of distant metastases was higher in the dabrafenib plus trametinib group [63].

A multicenter real-world study of the German Dermatologic Cooperative Oncology Group explored RFS, overall and melanoma-specific survival (MSS) and the response to subsequent treatment in 589 stage III melanoma patients undergoing adjuvant treatment with PD1 inhibitors or with BRAF+MEK inhibitors [64]. Among *BRAF*-mutant patients, RFS at 24 months was 49% for the PD1 group and 67% for patients treated with targeted therapy; 24-month MSS was 87% for the PD1 group and 92% for the targeted therapy group [64].

Roccuzzo and coworkers explored the real-life outcomes of adjuvant targeted therapy (dabrafenib plus trametinib) and anti-PD1 agents (pembrolizumab or nivolumab) in a group of 163 surgically resected melanoma patients at stage III (147 patients) or IV (19 patients) [65]. The large majority (93%) of patients treated with targeted therapy had *BRAF*-mutant melanomas. At 48 months of follow-up, both the immunotherapy and targeted therapy groups displayed comparable outcomes in terms of RFS (58.4% vs. 55.6%, respectively), DMFS (59.8% vs. 58.2%, respectively) and OS (69.5% vs. 62.4%, respectively) [65]. Therapy discontinuation due to adverse events was comparable in the two groups of patients.

Melanoma recurrence occurs in a significant proportion of melanoma patients undergoing adjuvant treatment with anti-PD1 agents; some of these patients displayed an early recurrence during anti-PD1 treatment, while other patients displayed a late recurrence occurring after the end of the treatment with anti-PD1 [66]. In a group of *BRAF*-mutant patients with recurrence after anti-PD1 therapy, Owen and coworkers reported a response to BRAF/MEK inhibitors in 18/23 patients with early recurrence and in 9/10 with late recurrence [61]. Bhave et al. reported 85 *BRAF*-mutant melanoma patients who developed recurrent disease after adjuvant treatment with BRAF/MEK inhibitors [67]. The response to anti-PD1, combination nivolumab/ipilimumab, BRAF/MEK inhibitor rechallenge, and ipilimumab monotherapy was 63%, 62%, 25% and 10%, respectively, and 2-year OS was 84%, 92%, 49% and 45%, respectively [67]. Taylor and coworkers explored a group of 73 *BRAF*-mutant melanoma patients who received adjuvant therapy with anti-PD1 agents and who experienced recurrence: all these patients underwent local therapy, and a group of these patients (61 patients) received a “second adjuvant” therapy with BRAF/MEK inhibitors, while a second group (12 patients) had no additional therapy [68]. RFS was significantly better among patients undergoing a second adjuvant treatment (30.8 vs. 4 months), but overall survival was similar in the two groups of patients [68].

As outlined above, randomized phase III clinical trials of adjuvant therapies have failed to show a significant benefit at the level of overall survival. In this context, a recent study presented at the ESMO Congress (Barcelona, Spain, 13–17 September 2024) reported the OS data of a large cohort of 1117 patients with stage III sentinel lymph-node-positive cutaneous melanoma, subdivided into two cohorts (one not receiving adjuvant treatments (506 patients) and the other one (611 patients) receiving adjuvant treatments with anti-PD1 or BRAF/MEK inhibitors); the 3-year OS rates were 80.9% and 80.1% in the cohorts of patients receiving adjuvant treatments or not, respectively [69].

Adjuvant immunotherapy and targeted therapy are costly and are both associated with the potential of life-long adverse events. Particularly, the studies on adjuvant anti-PD1 immunotherapy or targeted therapy with BRAF/MEK inhibitors have clearly shown a benefit at the level of RFS, but lack of OS benefit. This finding raises concerns about the long-term efficacy of adjuvant therapy in stage II/III melanoma patients. These observations have also shown that adjuvant therapy in melanoma patients is not a strong promoter of OS [70]. Additional major concerns include the adverse events induced by adjuvant therapies and their high cost [70].

Two studies have evaluated relative versus absolute benefit through the analysis of the number needed to treat (NTS, the number of patients required to receive an intervention in order to prevent one event of interest) and the number needed to harm in melanoma adjuvant therapy [71]. Projections for NNT among stage IIIA *BRAF*-WT patients increased by age from 14.78 (age from 40 to 44) to 142.86 (age 85 to 89), with patients in cohorts over 75 years having an NNT over 25 [72]. The cost per mortality avoided ranged from USD 2.75 million (age 40 to 44) to 27.5 million (age 85 to 89) [72].

Therefore, individualized decision-making is of crucial importance before the definitive incorporation of adjuvant targeted therapy and immunotherapy as standard treatments for melanoma patients [70]. An adequate risk stratification of melanoma stage II/III patients is required to guide treatment decisions for these patients. In this context, a recent study developed and validated a novel model to predict RFS and MFS after sentinel lymph node biopsy in stage II/III melanoma patients; the development cohort consisted of 4071 patients and the validation cohort consisted of 4822 patients. After the evaluation of a large number of potential predictors of melanoma-specific survival, six prognostic factors were retained for the elaboration of a final model: two clinical parameters (age at sentinel biopsy, primary tumor location) and four tumor-related parameters (sentinel node status, Breslow thickness, presence of ulceration and maximum diameter of the largest sentinel node metastasis) [73]. This model accurately predicted patient-specific risk probabilities for 5-year RFS and MFS, thus offering an important tool for clinical decisions to be adopted when evaluating adjuvant treatments in patients with high-risk melanomas.

### 3.3. Neoadjuvant Therapy of Melanoma

In addition to adjuvant treatments, other studies have attempted a different approach consisting of treating melanoma patients with resectable disease with immunotherapy or targeted therapy before surgical resection and then performing adjuvant treatment.

A pivotal phase Ib trial (NCT 02434354) on 30 stage III/IV resectable melanoma patients evaluated the long-term outcomes following a treatment based on neoadjuvant (a single dose of 200 mg of pembrolizumab) treatment 3 weeks before surgical resection, followed by 1 year of adjuvant pembrolizumab [74]. In an early report on this study, Huang et al. showed that 8 of 27 treated patients exhibited a complete response or major pathological response after neoadjuvant therapy: these rapid clinical responses were associated with the accumulation of exhausted CD8^+^ cells in the tumor at 3 weeks. Pretreatment immune signatures were associated with clinical response [67]. In contrast, immune suppression and mutational escape correlated with resistance to the treatment [74]. The follow-up of 30 patients at 61.9 months, treated in the context of this trial, showed the following: no deaths were observed among patients with complete or major pathological response, compared to a 5-year survival of 72.8% for the remainder of the cohort; 2 of 8 patients with major pathological response relapsed; 8 of 22 patients with incomplete pathological response relapsed; and the median time to recurrence was 3.9 years for patients with ≤10% viable tumors and 0.6 years for patients with >10% viable tumor cells [75].

The NeoCombi phase II trial (NCT 01972347) evaluated the safety and efficacy of neoadjuvant dabrafenib plus trametinib in 35 *BRAF*-mutant resectable stage IIIB-C melanoma patients; these patients received a treatment based on 12 weeks of neoadjuvant therapy, followed by 40 weeks of adjuvant therapy [76]. At resection, 86% of patients had a RECIST response, with 46% showing complete pathological responses (pCR) [76]. Grade 3-4 adverse events were reported in 29% of patients [76]. In a subsequent study, a long-term evaluation of these patients at 5 years was made. Overall RFS was 40%: 53% in patients with pCR and 28% in those with non-pCR; overall DMFS was 57%: 59% in patients with pCR and 55% in those with non-pCR; OS was 80%: 88% in patients achieving pCR and 71% in those not achieving pCR [76]. Overall recurrence was observed in 60% of patients: locoregional recurrence in 34% of patients and distant recurrence in 26% of patients [77].

The CombiNeo phase II trial evaluated dabrafenib and trametinib in 21 resectable IIIB-C or oligometastatic stage IV melanoma patients with *BRAF* mutations; patients were randomized to standard care (surgery and adjuvant therapy) or to neoadjuvant (4 weeks) and adjuvant therapy based on dabrafenib plus trametinib (44 weeks). The trial terminated early due to a markedly longer PFS and OS in the neoadjuvant group compared to the standard therapy group (for RFS, at 18.6 months, 71% vs. 0%, respectively; for OS, 19.7 months vs. 2.9 months, respectively) [78].

The OPACIN and OPACIN-Neo trials evaluated the safety and effectiveness of neoadjuvant therapy using nivolumab and ipilimumab in the treatment of high-risk stage III resectable melanoma patients [79]. In the OPACIN trial, 20 patients were randomized to receive adjuvant-only treatment based on four cycles of nivolumab plus ipilimumab, or neoadjuvant and adjuvant therapy based on two cycles of nivolumab plus ipilimumab [72]. The estimated 5-year RFS and OS rates for the neoadjuvant arm were 70% and 90% compared to 60% and 70% for the adjuvant arm [79]. The OPACIN-Neo trial evaluated different dosing schedules and identified that the most favorable was ipilimumab (1 mg/kg) combined with nivolumab (3 mg/kg) every three weeks, resulting after a follow-up of 47 months in 3 yr RFS and OS rates of 82% and 92%, respectively; for patients with a pathological response, the 3 yr RFS was 95% compared to 37% for patients not achieving a pathological response [79].

A personalized response-directed treatment after neoadjuvant treatment with nivolumab and ipilimumab was evaluated in the PRADO trial, an extension cohort of the OPACIN-Neo trial [80]. In this trial, patients achieving major pathological response after neoadjuvant therapy omitted surgical lymph node dissection and adjuvant therapy; patients with partial pathological response underwent surgical lymph node dissection only, whereas patients with pathological non-response underwent both surgical lymph node dissection and adjuvant therapy (according to their *BRAF* mutational status). The 24-month RFS and DMFS rates were 93% and 98% in patients with major pathological response, 64% and 64% in patients with partial pathological response, and 71% and 76% in patients with pathological non-response [80].

In 2021, the International Neoadjuvant Melanoma Consortium reported the first pooled analysis from six clinical trials of anti-PD-1-based immunotherapy or BRAF/MEK-targeted therapy involving a total of 192 melanoma patients [81]. A complete pathological response (pCR) was observed in 40% of patients (of these, 47% underwent targeted therapy and 33% immunotherapy). pCR correlated with improved RFS and OS. In patients with pCR treated with immunotherapy, RFS was 96%, but this was lower among patients who achieved pCR with targeted therapy (79%) [81]. A more recent analysis reported a pooled analysis on 818 patients with stage ≥IIIB melanoma (77% patients included in clinical trials and 23% real-world patients) [82]. Median follow-up was 3 years. Patients received neoadjuvant treatment with ICIs (610 patients: 169 PD1-alone, 351 PD1+ipilimumab, 59 PD1+LAG-3, 27 PD1+other immunotherapy agents), with targeted therapy (BRAF/MEK inhibitors, 88 patients), or with ICIs plus targeted therapy (120 patients) [82]. The analysis of 3-year EFS was as follows: 64% with PD1 alone, 76% with PD1+CTLA4, 82% with PD1+LAG-3, 37% with BRAF/MEK inhibitors, and 72% with targeted therapy plus PD1 [82].

The randomized SWOG S1801 clinical study evaluated two groups of melanoma patients with stage IIIB to IVC disease: one treated with three doses of preoperative pembrolizumab, followed by surgical resection and 15 post-operative pembrolizumab doses, and the other treated with adjuvant-only treatment with pembrolizumab [83]. After a median follow-up of 14.7 months, the neoadjuvant group comprising a total of 154 patients exhibited a significantly longer event-free survival (EFS) in comparison with the adjuvant-only group comprising a total of 159 patients (EFS at 2 years was 72% in the neoadjuvant group and 49% in the adjuvant-only group) [83]. The subanalysis of patients according to *BRAF* mutational status showed the following: in the *BRAF*-mutant group, EFS at 2 years was 74% in the neoadjuvant group and 55% in the adjuvant-only group [83]. This observation suggests a potentially better benefit for *BRAF*-mutant patients compared to *BRAF-WT* patients [76]. It is important to note that 16 patients randomized to the neoadjuvant treatment did not undergo surgery [75]. Pathology reports on surgical specimens showed that 21% of patients in the neoadjuvant arm displayed a complete pathological response [83].

Another recent study compared neoadjuvant and adjuvant therapy in melanoma patients with resectable disease. Thus, Blank et al. in the NCT 04949113 randomized phase III trial randomly assigned patients with resectable stage III melanoma to two cycles of neoadjuvant ipilimumab plus nivolumab followed by surgery, followed by 12 cycles of adjuvant nivolumab [84]. Only patients in the neoadjuvant group with a partial response or nonresponse received adjuvant treatment, which consisted of 11 cycles of nivolumab for *BRAF-WT* patients and dabrafenib plus trametinib for *BRAF*-mutated patients [84]. In the neoadjuvant group, 59% of patients had a major pathological response and 8% had a partial response [84]. The estimated EFS at 12 months was 83.7% for the neoadjuvant group and 57.2% for the adjuvant-only group; for the *BRAF^V600E^*- or *BRAF^V600K^*-mutant patients, the estimated EFS at 12 months was 83.5% in the neoadjuvant group and 52.2% in the adjuvant-only group; for the *BRAF-WT* patients, EFS was 83.9% in the neoadjuvant group and 62.4% in the adjuvant-only group [84]. Adverse events of grade 3–4 related to systemic treatment were experienced by 29% of the neoadjuvant group and 15% of the adjuvant-only group [84]. At 18 months of follow-up, EFS was 80.8% and DMFS was 85.7% in the neoadjuvant group, compared to an EFS of 53.9% and DMFS of 62.4% in the adjuvant-only group [85].

A sub-study 02C of the phase 1-2 KEYMAKER-U02 trial (NCT 04303169) is evaluating neoadjuvant pembrolizumab with or without investigational agents followed by adjuvant pembrolizumab for stage IIIB-C melanoma patients. This study involves five arms: arm 1, pembrolizumab plus vibostilimab (anti-TIGIT) (at 18 months, RFS 90% and EFS 81%); arm 2, pembrolizumab plus Gebasaxturev (Cosaxievirus A21) (at 18 months, RFS 90% and EFS 72%); arm 3, pembrolizumab alone (at 18 months, RFS 82% and EFS 80%); arm 4, pembrolizumab plus MK-4830 (anti-ILT4) (at 18 months, EFS 78%); arm 5, pembrolizumab plus favezilimab (anti-LAG-3) (at 6 months, RFS 93% and EFS 92%) [86].

### 3.4. Adjuvant Vaccination Studies

Several recent studies have explored different melanoma vaccination approaches in the adjuvant setting.

In this context, a recent study reported the clinical evaluation of multipeptide vaccines in melanoma patients in an adjuvant setting [80]. In an early study, vaccination with a cocktail of 12 melanoma peptides restricted by class I HLA molecules (12MPs) plus a tetanus toxoid helper peptide induced CD8^+^ cell response to these 12MPs in 100% of treated patients [87]. In an initial study, vaccination with a cocktail of 12 melanoma peptides restricted by class I HLA molecules (12MPs) plus a tetanus toxoid helper peptide induced CD8^+^ cel, with response to these 12MPs in 100% of treated patients [88]. Subsequently, a vaccine was developed comprising six melanoma peptides presented by class II HLA-DR molecules, whose injection induced CD4^+^ T-cell responses in most melanoma patients and induced a clinical response in some patients [89].

Starting from the two multipeptide vaccinations, a multicenter, randomized, phase II trial was developed to evaluate whether combined vaccination with 12MP and 6MP would enhance CD8^+^ T-cell response to the 12MPs and would improve clinical outcomes [87]. In this trial, a low dose of cyclophosphamide treatment was adopted to reduce regulatory T cells and to improve T cell response to vaccination [87]. The study involved the enrollment of four arms of patients: arms A+B treated with 12MP+Tetanoid Toxin Peptide (TTP), with arm B also pre-treated with cyclophosphamide; arms C+D with 12MP+6MP, with arm D also pre-treated with cyclophosphamide [80]. The analysis of long-term OS showed the following: median OS for 12MP+TTP was 12.9 years and for MP12+MP6 was not reached; OS rate estimates at 5, 10 and 15 years for MP12+MP6 were 74%, 68% and 61%, respectively, and for MP12+TTP were 68%, 56% and 45%, respectively [87]. For the individual study arms, the best RFS and OS data were observed for arm D, and the results were less favorable for arm A [87]. The most significant and durable benefit deriving from the 12MP-6MP vaccination was observed in male patients [87].

Recent studies based on the use of adjuvant dendritic cell therapy in stage IIIB/C melanoma patients failed to show a significant improvement over the placebo. Thus, in the MIND-DC randomized phase III trial, 148 patients with resected stage IIIB/C melanoma were randomized to adjuvant treatment with nDCs (autologous CD1c^+^ conventional and plasmocytoid dendritic cells loaded with tumor antigens) or a placebo [90]. After two years of follow-up, RFS in the group of patients treated with nDC was 36.8%, compared to 46.9% in the placebo group; median RFS was 12.7 months in the nDC group and 19.9 months in the placebo group [90]. In conclusion, this study provides evidence that adjuvant nDC treatment in stage IIIB/C melanoma patients may induce specific immune responses, but is not associated with clinical responses in terms of RFS [90].

mRNA-4157 is an mRNA-based cancer vaccine. When administered it will produce one of several dozen possible proteins commonly found in cancer patients. Particularly, mRNA-4157 targets up to 34 patient-specific tumor neoantigens to induce T-cell responses and potentiate antitumor activity. A recent randomized phase IIb study enrolled 157 completely resected stage IIIB-IV melanoma patients for adjuvant treatment with mRNA-4157 plus pembrolizumab (107 patients) or pembrolizumab monotherapy (50 patients). With a median follow-up of 23-24 months, RFS was longer with combination vs. monotherapy (HR 0.51). At 18 months, RFS and death event rates were 89% and 22% for the combination therapy compared to 62% and 40% for the monotherapy [91]. An updated analysis of the clinical results observed in the KEYNOTE-942 trial showed that, at a follow-up of 2.5 years, the RFS rate was 74.8% in the combo arm and 55.6% for pembrolizumab alone; in the combo arm, there was a 49% risk reduction in recurrence and/or death compared to pembrolizumab alone; there was a sustained improvement in DMFS in the combo arm versus pembrolizumab (HR 0.384); OS rate was 96% in the combo arm and 90.2% in the pembrolizumab arm; and RFS benefit was observed in tumor-burden-high and TMB-non-high melanomas [92]. Given the results obtained in the KEYNOTE-942 study, the INTerpath-011 randomized controlled trail was proposed, designed to evaluate the efficacy and safety of pembrolizumab plus mRNA-4157 versus pembrolizumab plus placebo in patients with high-risk stage II-IV melanoma [93].

The exploration of 4 resected non-small cell lung cancer patients and 12 melanoma patients undergoing treatment with mRNA-4157 alone (NSCLC) or in combination with pembrolizumab allowed the study of the mechanisms underlying the immunogenicity of mRNA-4157 [87]. mRNA-4157 induced neoantigen T-cell responses and the expression of cytotoxic CD8 and CD4 T cells. Particularly, mRNA-4157 induced consistent de novo and potentiated pre-existing T-cell responses to targeted neoantigens [94]. It is important to note that while mRNA-4157 is able to expand de novo T-cell clones, check point inhibitors act only on pre-existing T cells that may be suboptimally primed. The response of individual patients to mRNA-4157 immunotherapy was variable and associated with their pretreatment immunological status [94]. Pretreatment immunological status correlates with the response of T cells in patients treated with mRNA-4157 alone or in combination with pembrolizumab. Particularly, patients characterized by a high immune response exhibit a higher Th1/Treg ratio and are associated with a better clinical response, while those characterized by a low immune response display a lower Th1/Treg ratio and are associated with a lower clinical response [94]

### 3.5. Immunotherapy and Targeted Therapy of Metastatic Melanoma

Many studies have explored the safety and the efficacy of various immunotherapeutic treatments in melanoma patients with stage III/unresectable or metastatic disease (Table 3).

In this context, an international phase III, multicenter, randomized trial comparatively assessed nivolumab plus ipilimumab versus nivolumab alone or ipilimumab alone in unresectable/metastatic melanoma patients [88]. In total, 315 unresectable/metastatic III/IV melanoma patients were randomly assigned to one of these three treatments: nivolumab plus ipilimumab (nivolumab 1 mg/kg plus ipilimumab 3 mg/kg every 3 weeks, followed by nivolumab 3 mg/kg every 2 weeks); nivolumab 3 mg/kg every two weeks plus placebo; or ipilimumab 3 mg/kg for four doses [95]. Treatment was continued until disease progression or unacceptable toxic effects. Randomization was stratified according to BRAF mutational status, metastasis stage and PD-L1 expression in the tumor. A final analysis of this trial was carried out with a minimum follow-up of 10 years. The median OS was 71.9 months with nivolumab plus ipilimumab, 36.9 months with nivolumab and 19.9 months with ipilimumab [95]. The hazard ratio for death was 0.53 for nivolumab plus ipilimumab compared to ipilimumab alone and 0.63 for nivolumab compared to ipilimumab alone [95]. Median melanoma-specific survival was 120 months with nivolumab plus ipilimumab, 49.4 months with nivolumab alone and 21.9 months with ipilimumab alone [95]. Importantly, in patients with or without BRAF mutations, nivolumab plus ipilimumab significantly improved OS and MSS compared to ipilimumab alone [95]. It is important to note that, in patients with *BRAF^V600^* mutations, the difference in OS between nivolumab plus ipilimumab and nivolumab alone was more pronounced than in *BRAF-WT* patients (56% vs. 42% compared to 50% and 45%, respectively) [95]. The peculiar sensitivity of *BRAF^V600^* mutant melanoma to nivolumab plus ipilimumab seems to be related to over-representation in these tumors of interleukin-17 type helper T (T_H_17) gene expression signatures; high T_H_ 17 signatures and neutrophils predicted clinical responses to nivolumab plus ipilimumab but not to nivolumab alone or ipilimumab alone [96]. The presence of progression-free survival at 3 years predicted long-term survival [96]. Grade 3 or 4 treatment-related adverse events occurred in 59%, 23% and 28% of the patients in the nivolumab plus ipilimumab, nivolumab alone and ipilimumab alone groups, respectively [96].

Several studies have supported the efficacy of nivolumab plus ipilimumab in the treatment of melanoma patients with brain metastases. The phase II clinical study Check Mate 204 evaluated the safety and the efficacy of nivolumab plus ipilimumab in 94 melanoma patients with brain metastases [97]. The patients were treated with a protocol similar to that adopted by Wolchok et al. [95]. In this trial, 26% of patients achieved a CR and 30% a PR [90]. A long-term evaluation of this study involved a total of 119 melanoma patients with brain metastases, subdivided into a group of asymptomatic patients (101 patients) and a group of symptomatic patients (18 patients). Asymptomatic patients showed an ORR of 53.5% with a 36-month intracranial PFS of 54% and OS of 71.9%; in symptomatic patients, PFS was 18.9% and OS 36.6% [98].

The efficacy of nivolumab plus ipilimumab in the treatment of melanoma patients with brain metastases was confirmed in a real-world cohort of 79 patients, with an ORR of 46.9% and a CRR of 16.5%; during a 5-year follow-up, mOS was not reached [99]. The NIBIT-M2 phase III study confirmed these results, reporting a 4-year OS of 42.8% in melanoma patients with brain metastases [100].

Other studies have specifically addressed the treatment of melanoma *BRAF^V600^* mutant patients with brain metastases. The SECOMBIT phase II trial explored the optimal sequential treatment for metastatic melanoma patients: BRAF/MEK inhibitors as the first line followed by immunotherapy, or immunotherapy as the first line followed by BRAF/MEK inhibitors [101]. Thus, the Secombit trial involved the enrollment of 206 patients who were randomized across the three treatment arms: arm A (encorafenib plus binimetinib until progressive disease, followed by nivolumab plus ipilimumab); arm B (nivolumab plus ipilimumab until progressive disease, followed by encorafenib plus binimetinib); arm C “sandwich” (encorafenib plus binimetinib for 8 weeks, followed by nivolumab plus ipilimumab until progressive disease, followed by encorafenib plus binemitinib) [94]. At the 4-year of follow-up, the PFS rates for arms A, B and C were 29%, 55% and 54%, respectively; the OS rates at 3 and 4 years were 53% and 46% for arm A, 64% and 64% for arm B and 61% and 59% for arm C [101]. The results of this study clearly support the sequence of immunotherapy first followed by targeted therapy as the best therapeutic approach for metastatic melanoma patients [101]. In the SECOMBIT trial, the large majority of melanoma patients did not have brain metastases. Thus, the occurrence of brain metastases was explored during the follow-up of patients enrolled in the SECOMBIT trial: 23/69 in arm A, 11/69 in arm B and 9/68 in arm C [102]. At a median follow-up of 56 months, the 60-month brain-metastasis-free survival rates were 56% for arm A, 80% for arm B and 85% for arm C [102].

The DREAMSeq trial ECOG-ACRIN EA 6134 involved the treatment of metastatic *BRAF^V600^*-mutant melanoma patients with two different sequences of BRAF/MEK inhibitors and nivolumab/ipilimumab; patients treated first with nivolumab/ipilimumab and then dabrafenib/trametinib had a better PFS and OS compared to those treated first with dabrafenib and trametinib and then with nivolumab/ipilimumab [103].

The TROCOTEL trial, a multicenter, open-label, single-arm, phase II study, involved a cohort of *BRAF^V600^*-mutant melanomas and a BRAF-WT cohort; both groups of patients had melanomas with CNS metastases [97]. Patients with BRAF-mutant melanomas received atezolizumab, vemurafenib and cobimetinib, while BRAF-WT received atezolizumab and cobimetinib [104]. The cohort of BRAF-WT patients was stopped after the first 15 patients [97]. Intracranial ORR was 42% in BRAF-mutant melanomas and 27% in BRAF-WT melanomas [104].

The treatment based on nivolumab and ipilimumab was associated with a higher incidence of treatment-related adverse events, mainly induced by ipilimumab. Therefore, some recent studies have explored how to replace anti-CTLA-4 agents with anti-lymphocyte-activation gene 3 (LAG-3). In the phase II-III, double-blind, randomized RELATIVITY-047 trial, relatlimab (anti-LAG-3) and nivolumab as a fixed dose, compared with nivolumab alone, was evaluated in melanoma patients with unresectable or metastatic disease [105]. The mPFS was 10.1 month with relatlimab plus nivolumab and 4.6 months with nivolumab alone; at 12 months, PFS was 47.7% with relatlimab plus nivolumab and 36% with nivolumab alone [105]. Grade 3–4 adverse events were observed in 18.9% of patients in the relatlimab plus nivolumab group and in 9.7% of patients treated with nivolumab alone [105]. At 19.8 months of follow-up, the estimated OS was not reached among patients treated with relatlimab plus nivolumab and 34.1 months among patients treated with nivolumab alone [106]. Importantly, both *BRAF^V600^*-mutant and *BRAF-WT* melanoma patients benefited from relatlimab plus nivolumab treatment compared to nivolumab alone (HR 0.76 and 0.83, respectively) [106]. An update of this study at 3 years of follow-up continued to show a benefit of relatlimab plus nivolumab with regard to PFS, ORR, OS and MSS (melanoma-specific survival) [107].

The exploration of the immunological response to nivolumab plus relatlimib showed the following: higher IFN-γ level increases compared to baseline for nivolumab plus relatlimab compared to nivolumab alone [108]; decreased sLAG-3 levels in patients treated with nivolumab plus relatlimab [108]; higher baseline PD1^+^CD8^+^ and ICOS1^+^CD8^+^ T cells in responders to nivolumab plus relatlimab [109]; and a better response to nivolumab plus relatlimab and nivolumab alone in patients exhibiting higher LAG-3 tumor expression [109].

Long et al. performed an indirect treatment comparison between the RELATIVITY-047 and CheckMate 067 trial data using patient level data from each trial and reached the conclusion that the two different treatments used in these two different studies induced similar PFS, ORR, OS and MSS. A subgroup comparison showed larger numerical differences favoring nivolumab plus ipilimumab in acral melanoma and *BRAF*-mutant melanomas; nivolumab plus ralatlimab was associated with lower grade 3-4 adverse events compared to nivolumab plus ipilimumab [110].

The phase I/IIa, open-label RELATIVITY-020 trial part D assessed the safety and efficacy of nivolumab and relatlimab in 518 melanoma patients who had progressed during or within 3 months of 1 (D1) or >1 anti-PD1-containing regimes [111]. The ORR was 12% in D1 and 9.2% in D2; the median duration of response was not reached in D1 and 12.8 months in D2; mPFS was 2.1 months in D1 and 3.2 months in D2; the 6-month PFS rate was 29.5% in D1 and 27.7% in D2; grade 3–4 adverse events were experienced at a rate of 15% in D1 and 12.8% in D2 [111].

Relativity 048 is a phase I/II nonrandomized trial evaluating immuno-oncology triplets, including nivolumab+relatlimab+ipilimumab in various solid tumors. A recent study reported the preliminary results observed in a cohort of 46 melanoma patients with advanced disease. Median follow-up was 49.4 months, 8.7% had acral cutaneous melanoma, 50% were *BRAF*-positive, 79.9% were LAG-3 positive, 26.1% were tumor PD-L1-positive and 6.5% received prior adjuvant therapy. An ORR of 58.7% and an OS rate of 71.7% were observed [112]. Grade 3–4 adverse events occurred in 95% of patients [112]. Given the typology of the treated patients, this drug triplet seems to display an encouraging efficacy.

Other studies have explored new drug combinations for the treatment of patients with *BRAF*-mutant unresectable or metastatic melanomas.

Thus, Dummer evaluated spartalizumab (an anti-PD1 antibody) in combination with dabrafenib/trametinib in the treatment of *BRAF^V600^*-mutant unresectable melanoma patients. However, the results of this study failed to show any significant improvement of ORR and PFS rate in patients treated with spartalizumab plus dabrafenib/trametinib compared to dabrafenib/trametinib alone [113].

The Columbus trial involved the enrollment of 577 *BRAF^V600^*-mutant melanoma patients with unresectable metastatic disease randomly assigned to encorafenib plus binimetinib, vemurafenib or encorafenib [114]. Compared with vemurafenib, encorafenib plus binimatinib extended PFS (14.9 vs. 7.3 months) and mOS (33.6 vs. 16.9 months); the drug combination was well tolerated, and the rate of drug discontinuation was relatively low (10% vs. 14%, respectively) [114]. The data observed in this first report were confirmed in two other reports performed with a follow-up of 5 [115] and 7 years [116]. Particularly, at 7 years of follow-up, PFS and OS rates were 21.2% and 27.4% in the encorafenib plus binimetinib group and 6.4% and 18.2% in the vemurafenib group, respectively; median melanoma-specific survival was 36.8 months in the encorafenib plus trametinib arm and 19.3 months in the vemurafenib arm [116].

In part 2 of the phase III COLUMBUS trial, encorafenib (at 300 mg) plus binimetinib was compared to encorafenib alone (at 300 mg); the mPFS was 12.9 months for encorafenib plus binimetinib compared to 9.2 months for encorafenib; the ORR was 68% for encorafenib plus binimitinib and 51% for encorafenib alone [117].

The possible benefit deriving from an induction treatment with targeted therapy with BRAF + MEK inhibitors (encorafenib and binimetinib) prior to combined immunotherapy with nivolumab plus ipilimumab in patients with advanced *BRAF^V600^*-mutant melanoma was explored in the EORTC phase II randomized EBIN study [118]. However, the results of this study failed to show any significant improvement in PFS in the group of patients pretreated with encorafenib plus benitinib and with nivolumab plus ipilimumab, compared to those treated with nivolumab plus ipilimumab alone [118].

### 3.6. Adoptive Therapy with Tumor-Infiltrating T Lymphocytes (TILs) in Melanoma Patients Who Have Failed Immunotherapy and/or Targeted Therapy Treatments

Effective treatments are very limited for patients with advanced (unresectable or metastatic) melanoma who have progressed after immune checkpoint inhibitor treatment and targeted therapies.

Recent studies have shown that adoptive cell therapy with TILs has consistently shown efficacy in these refractory/relapsing melanoma patients. Adoptive cell therapy with TILs offers a potential therapeutic option for metastatic melanoma patients: this immunotherapy is based on the extraction of a fragment of tumor followed by expansion under culture conditions that are permissive for the expansion of a polyclonal population of T lymphocytes, allowing the generation of a large number of T cells to be infused back into the patients. The advantage of this therapy consists of the generation of T lymphocytes whose cytotoxic potential is potentiated during ex vivo expansion, and thus can address the large repertoire of individual neoantigens expressed on melanoma cells.

Two clinical studies have contributed to the approval of lifileucel (LN-144), an autologous TIL therapy that uses tumor-tissue T cells capable of recognizing tissue antigens and being expanded ex vivo, maintaining the heterogeneity of a repertoire of T cells using a centralized manufacturing process, for the treatment of metastatic melanoma patients. (Table 4) A phase II C-144-01 trial evaluated the safety and the efficacy of lifileucel in patients with advanced melanoma who had progressed on ICI and BRAF inhibitors. The first report of this study involved 66 patients infused with >1 × 10^9^ TIL cells; lifileucel induced a significant antitumor response with an ORR of 36%, with 2 complete responses and 22 partial responses, a disease control rate of 80% and a median duration of response not reached after 18.7 months of follow-up [119]. A later report on this trial involved 153 melanoma patients with ICI- or BRAF/MEK-resistant disease; treatment with lifileucel was associated 1-, 2-, 3- and 4-year os rates of 53%, 33.9%, 28.4% and 21.9% and with an ORR of 31.4%, with the median duration of response not reached [120]. The highest 4-year survival rates were observed in patients with more pronounced tumor responses (68.2%) [113]. The analysis of individual patients showed that few patients with brain or liver metastases or with high tumor burdens respond to treatment with TILs [120].

A second study (NCT 02278887) involved the evaluation of 168 melanoma patients with advanced disease (86% refractory to ICI treatment) to randomized treatment with ipilimumab or with autologous TIL (at least 5 × 10^9^ TILS); the infusion of TILs was preceded by lymphodepleting chemotherapy and followed by high-dose interleukin-2 infusions [114]. Median PFS was 7.2 months in the TIL group and 3.1 moths in the ipilimumab group; ORR was 49% in the TIL group and 21% in the ipilimumab group; and median OS was 25.8 months in the TIL group and 18.9 months in the ipilimumab group [121]. Grade 3–4 treatment-related adverse events were observed in 100% of patients undergoing treatment with TILs and 57% of those treated with ipilimumab [121].

In February 2024, lifileucel received accelerated approval by the FDA based on objective response rates and the duration of responses conferring substantial evidence of effectiveness in a population of melanoma patients with a high unmet medical need.

Given the results observed in the C-14401 trial, lifileucel was evaluated in melanoma patients with unresectable/metastatic disease, untreated with ICIs and treated with BRAF/MEK inhibitors if *BRAF*-mutated, in the context of the IOV-COM-202 clinical study [115]. In cohort 2A, 22 patients were enrolled and treated with a therapeutic regimen consisting of pembrolizumab, nonmyeloablative lymphodepletion, a single infusion of lifileucel (1 × 10^9^–150 × 10^9^ cells) until disease progression [122]. Of the patients, 36% had *BRAF* mutations. ORR was 63.6% (22.7% CR and 40.9% PR). At a median follow-up of 17.2 months, duration of response was not reached [115]. In a significant proportion of responding patients, responses were maintained for ≥12 months. The most common grade 3–4 adverse events were thrombocytopenia, neutropenia and anemia [122].

A recent study performed a systematic review and meta-analysis of the most relevant studies involving TIL therapy in advanced melanoma and reached several important conclusions: no difference was found in median OS between studies with prior anti-PD1 or anti-PDL1 treatment and without; ORR was 34% and 44% for the studies with and without prior anti-PD1 and anti-PDL1 treatment, respectively [123]. The pooled CRR was 10% [123]. These observations reinforce the evidence that TIL should be considered as a second-line treatment of choice for metastatic melanoma patients failing after anti-PD1/PDL1 therapy [123].

## 4. Conclusions

In the last two decades, there has been tremendous progress in the definition of the molecular alterations underlying the development of melanoma, one of the most malignant skin tumors. One of the most recurrent genetic alterations observed in melanoma is mutations of the BRAF protein kinase, whose constitutive activation plays a key role in the early stages of development of melanomas. The definition of these genetic alterations has fostered the development of new therapeutic approaches based either on the direct targeting of the mutated *BRAF* protein and of other constituents of the MAPK pathway or the targeting of the immune response, particularly at the level of immune check inhibitors. These two categories of drugs have led to a consistent improvement in the therapy of melanoma patients, *BRAF*-WT or *BRAF*-mutant, with an advanced stage of development. Particularly, this progress has led to a significant improvement in the OS of melanoma patients with unresectable or metastatic disease, with the definition of two immunotherapy treatments, based on the double targeting of PD1 and CTLA-4 or PD1 and LAG-3, that now represent the standard of care for these patients. The survival curves of these patients displayed stable tails that have plateaued over time, supporting the presumption that patients on the tail are in fact cured of their disease.

Treatment protocols based first on the use of immunotherapeutic agents and then of BRAF/MEK inhibitors are under evaluation and future studies will determine whether these switching therapy approaches may further improve the outcomes of these patients.

Patients progressing after anti-PD1-based therapy and targeted agents have limited therapeutic options. Therefore, there are no treatment options with approval based on data from patients with advanced melanoma who have progressed after one line of ICI therapy for BRAF-WT patients or two lines of therapy for *BRAF^V600^* mutation-positive tumors. However, recent studies have shown that some of these patients may significantly benefit from adoptive immunotherapy with TILs. Thus, lifileucel, a TIL-based therapy, was recently approved for these patients.

The risk of relapse and the prognosis of stage II and III melanomas with resectable disease are highly heterogenous, with a portion of patients having a consistent risk of relapse. Thus, numerous studies have explored the effect of adjuvant therapies based on ICIs or targeted therapy (in *BRAF*-mutant melanomas) and have shown a consistent improvement in PFS and RFS, but not OS, compared to the placebo. However, recent studies have shown that neoadjuvant therapy plus adjuvant therapy based on ICIs or targeted therapy may induce a significant therapeutic improvement compared to adjuvant therapy in terms of PFS and RFS, suggesting a possible improvement also at the level of OS. Furthermore, additional studies will be required to determine the optimal protocols of neoadjuvant/adjuvant therapy for *BRAF-WT* and *BRAF*-mutant stage II-III melanomas. In parallel, additional criteria for a better risk stratification of stage II-III melanoma patients have been defined and offer the tools for the selection of patients ideal for neoadjuvant/adjuvant treatments. In this context, it is important to note that neoadjuvant treatments offer an additional important parameter for the prognostic evaluation of stage II/III melanoma patients based on the pathological evaluation at the moment of tumor surgical resection. In fact, patients who achieve major pathological responses display unprecedented and lasting survival benefit, while those with partial or no responses show a lower survival benefit and will need alternative therapeutic approaches.

## Figures and Tables

**Table 1:** Adjuvant clinical studies involving stage II melanoma patients.

Clinical Trial	Patients	Treatment	RFS	DMFS	Safety	Parameters Correlating with Response
KEYNOTE-716 Phase III double-blind, randomized	976 stage IIB/C 487 (Pembro) 489 (Placebo)	Adjuvant pembroluzumab (PE) vs. placebo (PL)	36 months All 76.2%(PE) 63.4%(PL) IIB 79.7%(PE) 66.5% (PL) IIC 72.4%(PE) 58% PL)	36 months All 84.4%(PE) 74.7%(PL) IIB 86.7%(PE) 78.9%(PL) IIC 80.9%(PE) 68.1%(PL)	Grade 3–4 17.2%(PE) 5.1% (PL)	Not reported
CHECK MATE 76k Phase III double-blind, randomized	790 stage IIB/C 526 (Nivo) 264 (Placebo)	Adjuvant nivolumab (NI) vs. placebo (PL)	12 months All 89%(NI) 79% (PL) BRAF-WT 91.2% (NI) 77.1% (PL) BRAF-mut 87.3% (NI) 81.7% (PL)	12 months All 92.3%(NI) 86.7%(PL) IIC 87.9%(NI) 78.7%(PL)	Grade 3–4 10.3% (NI) 2.3% (PL)	Higher IFN-γ signature and % CD8^+^ cells

**Table 2:** Targeted therapy (dabrafenib plus trametinib) as adjuvant therapy in resected stage III *BRAF^V600^*-mutant melanoma patients.

Clinical Study	Patients	Treatment	RFS	DMFS	OS	Rate of Recurrence
Bai et al. Multicenter, retrospective cohort study	598 stage III BRAF-mutant melanoma	393 pts dabrafenib plus trametinib (DT) 205 pts anti-PD1 (PD1)	At 33 months DT 51 months PD1 44.8 months	Not reported	At 3 years DT 74.4% PD1 77.9%	Progression DT 45% PD1 27.7% Distant metastases DT 20% PD1 26%
Bloem et al. Dutch melanoma treatment registry nation-wide cohort	416 Two groups of 213 propensity score-matched stage IIIB BRAF-mutant patients	213 pts DT 213 pts anti-PD1	At 2 years DT 66.1% PD1 70.2%	At 2 years DT 84.1% PD1 82.1%	At 2 years DT80.4% PD1 85.1%	DT 34% PD1 30%

**Table 3:** Clinical studies in metastatic melanoma patients involving a long-term evaluation.

Clinical Trial	Patients	Treatment	PFS	OS	Safety	Parameters Correlating with Response
CHECK MATE 067 NCT 01844505, phase III, randomized	945 metastatic 314 (Nivo+Ipi) 316 (Nivo) 315 (ipi)	Nivo+Ipi Nivo Ipi Follow-up 10 years	Nivo+Ipi 11.5 mo Nivo 6.9 mo Ipi 2.9 mo	OS at 10 yr Nivo+Ipi 71.9 mo Nivo 36.9 mo Ipi 19.9 mo MSS Nivo+Ipi >120 mo Nivo 49.4 mo Ipi 21.9 mo	Grade 3–4 Nivo+Ipi 59% Nivo 23% Ipi 29%	BRAF-mut respond to Nivo+Ipi better than BRAF-WT; response to Nivo-Ipi is associated with TH17 signatures
CHECK MATE 204 NCT 02320058, phase II, open label, multicenter	119 with brain metastases 101 asymptomatic (cohort A) 18 symptomatic (cohort B)	Nivo+Ipi 36 months follow-up	36 mo intracranial Cohort A 57.4% Cohort B 18.9%	Cohort A 71.9% Cohort B 36.6%	Grade 3–4 5%	Not reported
SECOMBIT NCT 02631447, phase II, randomized	206 patients with BRAF^V600^ metastatic Arm A (69) Arm B (69) Arm C (68)	Arm A Enco+Bini→Nivo+Ipi Arm B Nivo+Ipi→Enco+Bini Arm C Enco+Bini 8wk; Nivo+Ipi→Enco+Bini	At 4 years Arm A 29% Arm B 55% Arm C 54%	At 4 years Arm A 46% Arm B 64% Arm C 59%	Not reported	Improved OS in patients with JAK mutations and low IFN-γ serum levels
RELATIVITY-047 NCT 03470922, phase II-III double blind, randomized	714 metastatic Nivo+Rela (355) Nivo (359)	Nivo+Rela Nivo	At 5 years Nivo+Rela 48.7% Nivo 39.4%	At 5 years Nivo+Rel 27.7% Nivo 21.6%	Grade 3–4 Nivo+Ipi 22% Nivo 12%	Improved response to Nivo+Rela in high baseline PD1^+^CD8^+^ and ICOS1^+^CD8^+^ T cells

**Table 4:** Adoptive therapy with TILs in melanoma patients with ICI refractory disease.

Clinical Trial	Patients	Treatment	PFS	OS	Safety	Parameters Correlating with Response
C-144-01 NCT 02360579, nonrandomized, phase II	153 advanced melanoma, ICI refractory	Lifileucel (autologous TIL) >1 × 10^9^ cells	ORR 31.4%	mOS 13.9 months 1 yr 54% 2 yr 33.9% 3 yr 28.3% 4 yr 22.2%	Grade 3–4 100%	Few responses in patients with high TMB and brain and liver metastases
M14 TIL NCT 092278887	168 advanced melanoma (86% ICI refractory) 84 TILs 84 ipilimumab (Ipi)	Autologous TILs At least 5 × 10^9^ cells	TIL 7.2 months Ipi 3.1 months	mOS TIL 25.8 months Ipi 18.9 months	Grade 3–4 TIL 100% Ipi 57%	Not Reported
